# Challenges in synthesising cost-effectiveness estimates

**DOI:** 10.1186/s13643-020-01536-x

**Published:** 2020-12-09

**Authors:** Gemma E. Shields, Jamie Elvidge

**Affiliations:** 1 Manchester Centre for Health Economics, Division of Population Health, Health Services Research, and Primary Care, School of Health Sciences, Faculty of Biology, Medicine and Health, University of Manchester, Manchester, M13 9PY UK; 2 Centre for Health Technology Evaluation, National Institute for Health and Care Excellence, Level 1A, City Tower, Piccadilly Plaza, Manchester, M1 4BT UK

**Keywords:** Meta-analysis, Systematic review, Cost-effectiveness analysis, Economic evaluation

## Abstract

Economic evaluations help decision-makers faced with tough decisions on how to allocate resources. Systematic reviews of economic evaluations are useful as they allow readers to assess whether interventions have been demonstrated to be cost effective, the uncertainty in the evidence base, and key limitations or gaps in the evidence base. The synthesis of systematic reviews of economic evaluations commonly takes a narrative approach whereas a meta-analysis is common step for reviews of clinical evidence (e.g. effectiveness or adverse event outcomes). As they are common objectives in other reviews, readers may query why a synthesis has not been attempted for economic outcomes. However, a meta-analysis of incremental cost-effectiveness ratios, costs, or health benefits (including quality-adjusted life years) is fraught with issues largely due to heterogeneity across study designs and methods and further practical challenges. Therefore, meta-analysis is rarely feasible or robust. This commentary outlines these issues, supported by examples from the literature, to support researchers and reviewers considering systematic review of economic evidence.

## Background

Cost-effectiveness analysis evaluates and compares the expected costs and health benefits of two or more healthcare interventions and supports decision-makers in assessing value for money. With growing and ageing populations, and an ever-expanding range of healthcare interventions, decision-makers face growing pressure to effectively distribute scarce resources.

Systematic reviews of economic evaluations allow readers to consider whether healthcare interventions have been demonstrated to be cost effective, uncertainty in the evidence base and key limitations of the evidence. The synthesis of economic evaluations in a systematic review most commonly takes a narrative approach, and subsequently, a query from non-health economists may be why a meta-analysis has not been considered or conducted, as this is the norm in systematic reviews with clinical outcomes. Version 5.1 of the Cochrane handbook for Systematic Reviews of Interventions discussed that there are no agreed methods for pooling estimates of cost effectiveness, but did not expand on the issues [1]. The most recent version of the handbook (version 6.0) does not discuss meta-analysis in the context of economic evidence [2]. A review found that out of 202 systematic reviews of health economic evaluations, only 3 used quantitative synthesis [3]. Although rare, there are examples of systematic reviews of cost-effectiveness studies in the recent literature that have conducted a meta-analysis, so to non-health economists, it may not be clear why meta-analysis is not routinely conducted.

This paper aims to briefly summarise why conducting a meta-analysis of economic evaluation outcomes, focusing on incremental cost-effectiveness ratios, costs, and quality-adjusted life years, is usually inappropriate. Systematic reviews by the authors and the wider literature act as case studies to illustrate the discussed problems in practice [4].

## Key complications in synthesising cost-effectiveness outcomes

In the sections below, we detail the key factors that contribute to a synthesis of cost-effectiveness outcomes being inappropriate. Note that this is not a critique of economic evaluation; the nature of cost-effectiveness analysis, particularly decision-analytic modelling, often necessitates combining data from different sources and assumptions to be made by the analyst. This is often done well, including a detailed exploration of the uncertainty. However, it does create challenges when synthesising across the wider evidence base.

### Heterogeneity

A recent review published in this journal discussed the substantial heterogeneity across identified economic evaluations for stress urinary incontinence [5], which is a common conclusion of systematic reviews of economic evaluations. There are multiple sources of heterogeneity across economic evaluations, which will limit the usefulness of a synthesis of the evidence. We discuss some of the key problematic areas below. Note that some of these areas will be familiar to researchers who conduct meta-analysis of clinical outcomes, such as population variability across populations, included interventions and outcomes [2, 6]. However, the synthesis of multiple sources of evidence and variation in methods used across economic evaluation introduces further uncertainty (such as structural heterogeneity).

#### Populations and samples

A key factor of heterogeneity in economic evaluations is variation in the populations considered and samples informing data. Economic evaluations imbedded into trials or observational studies will focus on a sample of the population meeting a specific inclusion criterion. Economic models also concentrate on a specific patient population but often rely on identifying a data from multiple existing sources which will likely come from different samples of patients. Therefore, economic evaluations identified in a review are expected to have been informed by data from samples with different characteristics (e.g. demographics, aetiology). These characteristics are likely to impact on the inputs (e.g. baseline risk) and, potentially, the subsequent results. In a review of economic evaluations for the population with schizophrenia, the precise definition of schizophrenia differed across studies, some patient characteristics varied, and others were not presented consistently [4]. Overall, researchers may struggle to restrict a meta-analysis to a specific and well-defined population group and may not have enough information to do so confidently.

#### Alternatives

Economic evaluation typically compares an intervention with current best practice or usual care, which may vary by setting. Analyses will be less comparable if they include different comparators. If an analyst were interested in synthesising all cost-effectiveness results for a disease with more than two alternatives treatments, they may identify economic evaluations making various comparisons which might necessitate a network meta-analysis. Furthermore, a non-specific or multicomponent comparator like ‘best supportive care’ might look very different across settings, in both the care provided and who receives it. In a review of economic evaluations for schizophrenia and bipolar disorder, the most common comparator used was standard care; however, its definition and the level of detail provided varied greatly between studies, reducing comparability [4].

#### Structural and methodological

Many of the sources of heterogeneity are associated with the synthesis of evidence and/or measurement and valuation, but potential heterogeneity across the methods used is equally important to consider. Structural heterogeneity occurs when analysts make different decisions and assumptions whilst developing their cost-effectiveness evaluations [7]. Not only are there considerable differences between modelling studies and analyses performed alongside a trial (or observational study), but within each type analysts have many design choices available to them. Modelling studies can use different structures (e.g. decision trees, state-transition models, patient simulations) which should theoretically yield the same conclusion about the cost effectiveness of an intervention, however, due to the diverging use of data and assumptions made results are likely to differ. For example, in oncology, published examples have demonstrated that different model structures estimate different durations spent in key health states, impacting cost-effectiveness results [8–12]. Despite this, in the same disease area, justification for the choice of model structure is often minimal [13]. For cost-effectiveness analyses alongside trials, there are many methodological differences that may contribute to variation in results. These include, but are not limited to, analysis approach (e.g. intention-to-treat), methods to account for missing data (e.g. imputation techniques) and regression models used [14].

A further example of structural heterogeneity is the choice of time horizon, which, for an economic model, should be long enough to capture all-important differences in outcomes between the interventions being compared [15]. This is open to interpretation which can lead to different models using different time horizons. Modelling a ‘lifetime’ duration could yield very different cost-effectiveness estimates to an analysis conducted alongside a trial, which is likely to use a shorter timeframe. In a published review of economic evaluations for cardiac rehabilitation the modelled time horizons ranged from 5 months to lifetime [16].

Economic evaluations also typically use discount rates to estimate the present-day value of costs incurred and health accrued in the future. Higher discount rates place less value on future costs and health gains, meaning otherwise identical economic evaluations will generate different results if they use different discount rates. Different settings require different discount rates. For example, the National Institute for Health and Care Excellence (NICE) in England requires future outcomes to be discounted by 3.5% per year [17]. The Canadian Agency for Drugs and Technologies in Health (CADTH) uses a lower rate of 1.5% per year, whilst the Health Information and Quality Authority (HIQA) in Ireland uses 4% [18, 19]. The discount rate used by an economic evaluation is therefore often defined by the relevant health technology assessment body for its setting. However, even when a discount rate is recommended in a specific country, adherence varies [20].

#### Data collection methods

Inconsistent data collection methods, for data that inform costs and health outcomes, may also limit the comparability of cost-effectiveness estimates. For example, if one study followed up its participants regularly and over a long time, it would inform the change in outcomes from baseline more accurately than a study with few or infrequent follow-up points. Service use data can be collected using self-report questionnaires or routinely collected data, which vary in terms of reliability and completeness [21]. This applies directly to economic evaluations conducted alongside effectiveness studies, and indirectly to modelling analyses if the primary evidence sources used different data collection methods.

#### Effectiveness evidence

Economic evaluations across a range of countries may be informed by limited effectiveness data. In an example cost-effectiveness review of influenza vaccination for the older population, some studies reported using effectiveness evidence data from other countries to estimate the health outcomes associated with vaccination in their own setting [22]. Synthesising these studies may mean the common, limited evidence base has a disproportionate influence on the final results [23]. This problem may be likely in the case of cost-effectiveness analysis, where it is common to develop one model and adapt it to a range of countries using similar data. Where evidence is specific to one setting or point in time, synthesising across studies will ignore issues with external validity.

#### Health outcomes

A widely used summary measure used in health economics is the quality-adjusted life year (QALY) which combines morbidity and mortality into a single measure and has the advantage of being used across a range of treatments and settings [24]. Its common use increases the likelihood of identifying comparable cost-effectiveness analyses. However, variation in the methods used to derive QALYs may still make different studies unsuitable for synthesising. Firstly, to generate QALYs, studies need health utilities, which represent strength of preferences for a health state. There is substantial variation in the available methods to calculate utilities, including direct and indirect measurements. Direct approaches include preference-based standard gamble and time trade-off methods and the non-preference-based visual analogue scale. Indirect methods involve the collection of data from generic or disease-specific health status measures, and the application of preference weights indicating the relative importance of each aspect of health status. There is considerable variation in health status measures (e.g. the Short Form 36, the Health Utilities Index, or EuroQoL-5D questionnaires) in the amount and depth of information they elicit to establish a person’s state of health [25]. Further, preference weights differ according to whose preferences are measured (e.g. which country, patients, general public) and the methods used to generate them (e.g. time-trade off or discrete choice experiment). The literature comparing these methods has demonstrated that the method used affects the utility values [26–31]. QALYs also account for length of life, incorporating mortality estimates, which vary by setting and over time. All these factors reduce the comparability of QALY estimates. In an example review of the cost effectiveness of cardiac rehabilitation, heterogeneity in utilities is demonstrated as across the identified cost-utility studies, three use generic indirect questionnaires, one uses a disease-specific indirect questionnaire and two studies used direct methods (time trade-off) [16].

Away from the QALY, there are many disease-specific health outcomes that can be used in cost-effectiveness analysis, but if evaluations use different measures of effectiveness their results cannot be meta-analysed. For example, a review of psychological interventions for schizophrenia identified some outcomes that were only used in a single study (e.g. vocational recovery) [4].

#### Costs and resource use: perspective used

Significant heterogeneity can exist in the resource use and unit cost inputs used in economic evaluations. Different analyses may use different perspectives for costs, which dictate what resources should and should not be compared to evaluate cost-effectiveness. For example, analyses considered by NICE take a payer perspective, focusing on costs incurred by the NHS and social care system [17, 32]. Other perspectives may include different categories of resource use that capture broader effects. For example, the National Health Care Institute (ZIN) in The Netherlands requires that evaluations use a societal perspective, including costs associated with employment, informal care and other sectors such as education [33]. Other settings may use more limited societal approaches that require only a few, specific additional resource use items [34]. Any societal perspective will almost certainly lead to different total cost estimates compared with a payer perspective, and potentially different incremental costs and cost-effectiveness results.

#### Costs and resource use: values used

Even if a common perspective is used between studies, there may be differences in what resources different health systems provide, or authors may make different decisions about including or excluding specific costs [34]. If different types of resource use are included, cost results will not be comparable. Furthermore, there may be differences in the unit costs used to value resources due to how and when they are collected. For example, to identify a unit cost for a procedure, one study may use a nationally available average value whilst another uses detailed cost data collected by centres participating in a trial. These approaches are unlikely to generate the same cost estimate. In an example review, focusing on the cost effectiveness of interventions for postnatal anxiety and depression, of the 8 studies identified, only half had the same perspective (health and social care) [35]. In the same studies, price years ranged from 1992 to 2014, meaning an analyst would need to standardise to account for inflation. Additionally, a clear source of cost heterogeneity occurs when economic evaluations do not use a common currency. To synthesise cost results, an analyst would need to apply a conversion, using an appropriate exchange rate.

### Practical issues

Even if the myriad sources of heterogeneity between modelling studies are not expected to have a significant impact on the comparability of cost-effectiveness estimates, an analyst seeking to synthesise results would face further practical obstacles. Firstly, whilst the sample size is often used to weight the impact of each study included in a meta-analysis, there is no obvious equivalent to assign weights to modelling studies. This may rely on a subjective assessment of study quality, and how that would translate to a numeric weight is unclear.

It is good practice for economic evaluations to investigate uncertainty [7, 36]. Modelling studies frequently report probabilistic results, reflecting parameter uncertainty. Likewise, trial analyses will often be bootstrapped to generate thousands of net pairs of costs and health outcomes. Both approaches help to embed parameter uncertainty in an average measure of cost effectiveness. Some studies may report only deterministic results; however, a deterministic incremental cost-effectiveness ratio (ICER) may be an inaccurate estimate of the ‘true’ ICER [37], and would therefore be inappropriate for meta-analysing. If ‘one-way’ sensitivity analyses are reported, the deterministic results may not be comparable, as different distributions or ranges may have been used to vary the parameter values. When probabilistic results are reported, it is uncommon for modellers to provide evidence that the model has been simulated enough times for the average ICER to approximately converge on its asymptote. If convergence has not been demonstrated, random noise (i.e. Monte Carlo error) might not have been minimised; therefore, the probabilistic ICER may still be an inaccurate estimate [38]. These variations in how final ICERs are reported to add to the challenge of choosing which (if any) results can be synthesised robustly.

The ICER itself can be challenging to interpret. Being a ratio, an ICER can be very high when the denominator (e.g. incremental QALYs) is small, and thus is highly sensitive to small changes in the denominator. This can lead to spurious conclusions about strategies that are essentially equally effective. Further, a synthesis of cost-effectiveness results may be disproportionately influenced by a study that happens to produce a very high ICER. A negative ICER is even more problematic because they are ambiguous; it could mean an intervention is cheaper and more effective than its comparator, or more expensive and less effective. Therefore, negative ICERs should not routinely be presented [39]. Furthermore, if an intervention is less effective but less costly than its comparator, the ICER represents the cost saving per QALY lost by using the less effective intervention. Here, counterintuitively, a higher ICER indicates that the intervention of interest is more cost effective. These inconsistencies can make ICERs inappropriate for synthesis. Net benefits are easier to interpret, but introduce a new issue; they cannot be calculated without defining a specific cost-effectiveness threshold, or maximum allowable cost per QALY gained, whereas an ICER can be calculated independently of the threshold. All else equal, the net benefit (cost-effectiveness) of an effective intervention will be higher if the threshold is higher. Net benefit results using a common cost-effectiveness threshold could be synthesised, though this may be complicated by different currencies and price years as discussed above.

It may be more appropriate to meta-analyse incremental cost and incremental QALY results separately, then calculate the synthesised cost-effectiveness estimate from those results. However, even when doing so, an analyst should be mindful of different approaches to reporting such results. Incremental costs and QALYs are often reported per patient, but it is not uncommon to see aggregate results presented for the full trial or modelled population, the size of which may be unclear. For an ICER calculation, this distinction does not matter, but for synthesising incremental outcomes separately a common denominator should be used. Further, the baseline strategy (e.g. standard care or no intervention) against which incremental costs and QALYs are estimated should be common. Studies might do this, or they might present ‘fully incremental’ results, comparing all options with each other simultaneously, such that an analyst may need to calculate the incremental results of interest needed for synthesis [40, 41]. Furthermore, others might present neither the incremental results of interest nor sufficient detail for an analyst to do so [42].

## Conclusions

The text and examples above present an overview of some of the significant heterogeneity and practical challenges that mean a meta-analysis following a systematic review of economic evaluations is rarely feasible or robust. It is the opinion of the authors that the large number of challenges described will almost always be insurmountable, such that a meta-analysis of cost-effectiveness estimates would not provide meaningful results. The sections above will help researchers conducting reviews of economic evaluations to explain why it is unsuitable to conduct a meta-analysis, as well as assisting students and reviewers in understanding the issues. Note, there are four key types of economic evaluation; cost-benefit, cost-effectiveness, cost-utility and cost-minimisation analyses [43]. Due to the predominance of cost-effectiveness analysis (of which cost-utility is a subset) we have focused on outcomes related to these types of analysis [44]. If other types of analysis are included in a review, the challenges (in particular, heterogeneity) will grow further.

There are some tentative suggestions for when synthesis may be plausible, such as studies with similar designs, settings, perspectives and time horizons. However, comparable economic evaluations are rarely conducted in the same setting. Further, reviewers considering the heterogeneity and practical challenges likely to impact a synthesis will be restricted by the quality of reporting and the information presented in a paper. For some issues (e.g. related to the source of the effectiveness evidence), this may mean going back to original studies for further details. If synthesis is attempted, careful consideration should be given to which results are synthesised; it may be the case that probabilistic (and converged) incremental costs and health outcomes should be meta-analysed separately, if reported, and then used to calculate a synthesised cost-effectiveness estimate. Future research could consider producing a framework (or checklist) for when a synthesis might be robustly performed and useful to decision-makers. However, it is recognised that heterogeneity is likely to always limit the usefulness of a synthesis as differing settings, objectives and audiences for cost-effectiveness analysis will result in justifiable differences between studies. In general, the authors consider that for the purpose of informing health care resource allocation, decision-makers should focus on the most applicable studies to their setting. The generalisability of economic evidence to the specific decision-making context has been regarded as even more important than the methodological validity of the analysis [45]. In the absence of generalisable results, the possibility of conducting further economic evaluation should be explored, rather than synthesising less applicable results and increasing the risk of decision error.

As noted in the introduction, there are existing publications that have conducted a meta-analysis following a systematic review of economic evaluations. This paper is not suggesting that they were wrong to do so; rather, that there are extremely limited circumstances where it is appropriate and informative. We do note that novel methods are being explored in this area [46, 47]. Furthermore, more consistent methodologies (e.g. through researchers following best practice guidelines) and adherence to reporting standards may make it easier in the future to judge whether synthesis is feasible.

Finally, it should be noted that narrative reviews of economic evaluations remain a very useful undertaking for a range of audiences; for example, they can help researchers who aim to identify evidence or inform approaches to analysis, and decision-makers to identify evidence most relevant to their objective and setting [3]. This commentary may support narrative synthesis by helping reviewers to identify why cost-effectiveness estimates may differ between studies and to provide a useful guide of key features to compare.

## Data Availability

Not applicable.

## Abbreviations

CADTH: Canadian Agency for Drugs and Technologies in Health
HIQA: Health Information and Quality Authority
ICER: Incremental cost-effectiveness ratio
NICE: National Institute for Health and Care Excellence
QALY: Quality-adjusted life year
ZIN: National Health Care Institute (Zorginstituut Nederland)
